# Spatiotemporal Overlap and Scent-Marking Behaviour of the European Badger and the Stone Marten in a Mediterranean Landscape

**DOI:** 10.3390/ani16193041

**Published:** 2026-09-27

**Authors:** Francesca Trenta, Rebecca Mamola, Andrea Gallizia, Claudio Capasso, Matteo Ferretti, Adriana Vallesi, Adriano De Ascentiis, Giampaolo Pennacchioni

**Affiliations:** 1 Informal Research Group, Centre for Studies on Ecology and Biodiversity of the Apennines (CSEBA), Contrada Vallonica 11, 62010 Treia, Italy; rebeccamamola18@libero.it (R.M.); gallizia.andrea66@gmail.com (A.G.); c.capassol96@gmail.com (C.C.); matteoferretti18@gmail.com (M.F.); gp.pennacchioni@libero.it (G.P.); 2 School of Biosciences and Veterinary Medicine, University of Camerino, 62032 Camerino, Italy; adriana.vallesi@unicam.it; 3 Natural Reserve Oasi WWF Calanchi di Atri, 64032 Atri, Italy; adriano.deascentiis@gmail.com

**Keywords:** *Martes foina*, *Meles meles*, camera trapping, mesocarnivores, spatiotemporal patterns, scent marking behaviour, wildlife ecology

## Abstract

To investigate how *Meles meles* and *Martes foina* share a relatively restricted and heterogeneous Mediterranean environment, we analyzed space use, temporal patterns, and chemical communication of the two mesocarnivores in the WWF “Calanchi di Atri” Regional Nature Reserve (Abruzzo, Italy) over a period of 12 months (August 2022–July 2023), using ten infrared camera traps along fixed transects. For each record, date, time, GPS location, and scent-marking behaviour were annotated. A total of 765 records were documented, of which 595 and 170 referred to *M. meles* and *M. foina*, respectively. Spatial analyses revealed similar patterns of territorial use by the two species, while temporal patterns differed slightly. The detection rate of *M. meles* peaked in central night-time hours, while *M. foina* extended into crepuscular periods. Scent-marking was more frequent and ritualized in *M. meles*, peaking in winter and spring, whereas *M. foina* exhibited opportunistic marking. These differences in temporal patterns and communication behaviours may thus contribute to reducing potential interactions between the two mesocarnivores in a shared, fragmented Mediterranean habitat.

## Simple Summary

This study explored whether two small European carnivores, the European badger and the stone marten, share the same habitat by analyzing their spatiotemporal distribution and marking behaviour. The research was carried out in a protected area of central Italy where the presence of the two species had not previously been investigated, using camera traps over a one-year period. The results showed similarities in spatial use, whereas nocturnal activity patterns differed slightly, with badgers being more active during the darkest hours and stone martens showing greater activity around twilight. The badger uses scent signals more frequently and in a structured and systematic way, especially during winter and spring, whereas the marten exhibits this behaviour more occasionally. The comparison of the spatial and temporal patterns of detections and communication strategies of the two mesocarnivores provides insights into how wildlife coexist in complex environments and offers useful information for biodiversity conservation.

## 1. Introduction

Understanding the mechanisms that allow ecologically similar species to coexist is a central topic in community ecology. In carnivore assemblages, coexistence is often mediated by niche partitioning along spatial, temporal, and trophic dimensions, which reduces direct competition and promotes resource sharing among sympatric species [1,2,3,4]. In Mediterranean ecosystems, where habitat heterogeneity and anthropogenic disturbance frequently shape species distributions, such mechanisms are particularly important for maintaining mesocarnivore diversity [5,6,7].

Among European mesocarnivores, the European badger (*Meles meles* Linnaeus, 1758) and the stone marten (*Martes foina* Erxleben, 1777) represent a suitable model for investigating coexistence dynamics. Both species are ecological generalists characterized by broad trophic niches and substantial behavioural flexibility, allowing them to exploit a wide variety of natural and human-modified habitats [8,9,10,11,12,13]. Nevertheless, they differ in several aspects of their ecology. The European badger typically exhibits strong site fidelity, well-developed territorial systems, and extensive use of scent-marking behaviour in social communication [14,15]. In contrast, the stone marten is generally considered more flexible in habitat use and spatial behaviour, frequently occurring in agricultural and peri-urban environments and displaying less rigid territorial organization [12,16,17].

Reproductive phenology may strongly influence seasonal activity and communication patterns in both species. The European badger exhibits a complex reproductive strategy characterized by delayed implantation and mating opportunities throughout much of the year, with reproductive peaks occurring in late winter–spring and summer–autumn. Cubs are generally born between January and March and remain within the sett during the first weeks of life. Scent-marking behaviour is known to increase during reproductive periods, when chemical signals play an important role in mate advertisement and social interactions [18,19,20,21]. In contrast, the stone marten mates mainly during summer (June–August), followed by a prolonged period of delayed implantation. Implantation occurs in late winter, and births generally take place between late March and early April, with females remaining associated with setts while rearing young [22,23,24]. Consequently, seasonal variation in activity and marking behaviour may partly reflect reproductive constraints and communication requirements in addition to environmental and trophic factors.

Although the two species differ in behavioural ecology, they may overlap substantially in resource use and habitat exploitation. Previous studies conducted in Mediterranean landscapes have documented both spatial overlap and varying degrees of segregation between badgers and martens, suggesting that coexistence is facilitated by a combination of spatial, temporal, and behavioural adjustments rather than strict habitat separation [3,7,25,26]. While temporal patterns and habitat use have received considerable attention, the potential role of chemical communication in mediating coexistence remains comparatively understudied. In the European badger, scent-marking behaviour has been extensively investigated and is known to vary according to season, social organization, and territorial context [15,27,28]. By contrast, information regarding scent-marking behaviour in the stone marten remains limited, particularly in Mediterranean environments, and comparative studies evaluating marking behaviour alongside spatial and temporal activity patterns are still scarce [23].

To study the coexistence dynamics between *M. meles* and *M. foina* in Mediterranean landscapes, we planned to analyze the spatial use, temporal pattern, and scent-marking behaviour of these two mesocarnivores living in the “Calanchi di Atri” WWF Regional Nature Reserve. This protected area represents an ideal setting for investigating these processes due to its high environmental heterogeneity, characterized by badlands, woodland patches, grasslands, cultivated fields, and ecotonal habitats. Such landscape complexity provides suitable conditions for the coexistence of mesocarnivore species while potentially promoting behavioural adjustments associated with resource partitioning. No information regarding population density and setts of European badger and stone marten in the Reserve was available; therefore, this study provides a baseline for assessing the local presence and distribution of the two mesocarnivores in the study area. The method of choice was camera trapping, which is widely used in wildlife ecology as a non-invasive approach that provides continuous, standardized observations while minimizing disturbance to animals [29,30,31,32]. This method is particularly valuable in fragmented Mediterranean landscapes, where habitat mosaic variety may strongly influence species distribution, activity patterns, and interspecific interactions [5,7]. With this approach, we were able to: (i) describe seasonal and nocturnal activity patterns of both species; (ii) assess the degree of spatial and temporal overlap between them; and (iii) compare the distribution of scent-marking behaviour across space and time. The integration of the results obtained in this study offers new insights into the ecological processes that allow two widely distributed European mesocarnivores to coexist within the same geographical area.

## 2. Materials and Methods

### 2.1. Study Area

The study was carried out within the WWF Oasis “Calanchi di Atri” Regional Nature Reserve (Abruzzo, Italy), established in 1995 in an area previously designated by the European Community as a Site of Community Importance (SCI IT7120083) (Figure 1a,b). In 1999, the site was formally recognized as a WWF Oasis.

The reserve covers approximately 600 ha and is characterized by a heterogeneous habitat mosaic including badlands, woodland patches, grasslands, ponds, gullies, and agricultural areas. This environmental heterogeneity supports diverse vertebrate communities and provides suitable habitat for several mesocarnivore species (Figure 1c) [6,23,25,33].

### 2.2. Data Collection

Monitoring was conducted over a 12-month period (August 2022–July 2023) using a standardized passive camera-trapping protocol. A total of 10 infrared digital camera traps were positioned along fixed transects within the Reserve (Figure 2). Sampling locations were selected to encompass the main habitat types present within the study area, including badlands, forest edges, agricultural areas, and ecotonal corridors, in order to capture the heterogeneity of landscapes used by mesocarnivores [29,30,31,32].

The camera traps consisted of ten CW-200W01 devices equipped with 2 MP CMOS sensors and configured for video-based wildlife monitoring. Each unit was programmed to record 30 s videos per trigger event.

Camera traps were set to monitor activity between sunset and sunrise (7:00 p.m.–07:00 a.m.), at an average height of 80–100 cm above ground level. Camera traps were oriented along natural trails, vegetation edges, and constrained passage points, as recommended by established guidelines to optimize detectability of medium-sized carnivores [30]. Geographic coordinates of camera-trap stations were recorded and subsequently verified using Google Earth Pro, allowing the association of latitude and longitude data with specific landscape features. These locations were kept constant throughout the study period to ensure temporal comparability and minimize spatial bias. Camera-trap placement followed a targeted sampling strategy aimed at maximizing detection probability, focusing on ecotonal areas, agro-pastoral habitats, Mediterranean scrub, and frequently used wildlife passages identified through field signs and habitat suitability rather than proportional habitat representation [29,30,31].

To maintain consistent sampling effort, camera traps were checked on a biweekly basis to replace batteries and memory cards, and to remove any vegetation that could obstruct the field of view or cause false triggers.

The overall protocol followed the internationally recommended standards for ecological studies based on camera trapping, including criteria of repeatability, comparability among sites, and minimization of disturbance to monitored animals [29,31,34]. Individuals of *M. meles* and *M. foina* were identified from camera-trap videos based on their observable phenotypic characteristics, including body size, coat coloration, and other external morphological traits. The identification of *M. foina* was also considered in light of the absence of pine marten (*M. martes* Linnaeus, 1758) detections throughout eight years of camera-trap monitoring in the study area.

### 2.3. Data Processing

Data generated by camera traps were downloaded at regular intervals and organized into electronic spreadsheets (Microsoft Excel), following a standardized structure to ensure data traceability and quality. Each trigger event was considered an independent record only when it occurred at least two minutes after the previous event. Data included detected species, exact date and time of the event, and GPS location of the sampling point. As required by international camera-trapping protocols, the recorded coordinates refer to the camera trap location rather than the animal’s position, since the device represents the spatial sampling unit [30,31,35]. This approach enables consistent spatial analyses and reduces georeferencing errors related to subject position within the frame.

For each event, scent-marking behaviour, such as feces or urine deposition, grooming on surfaces, ground scratching, lateral body rubbing, or muzzle rubbing, was also annotated. Additional data quality checks included the removal of false triggers caused by wind or other non-biological factors, verification of temporal metadata (synchronization of timestamps across devices), and manual review of video recordings in cases of uncertain species identification. False triggers and uncertain records were excluded prior to analysis, following recommendations by Burton et al. and Caravaggi et al. [29,32].

### 2.4. Statistical Analyses

All statistical analyses were performed in R (version 4.5.3) through RStudio (version 2026.01). Since the number of detections differed substantially between species and the data did not meet the assumptions of normality, non-parametric approaches were adopted throughout the study.

To compare the relative use of camera-trap stations by *M. meles* and *M. foina*, the number of detections recorded at each station was converted into proportions of the total detections for each species. Camera-trap stations were treated as paired sampling units, and differences in proportional use between species were assessed using Wilcoxon signed-rank tests. The same approach was applied to compare the proportional distribution of scent-marking events between species.

Associations between species in the spatial distribution of detections and scent-marking events were evaluated using Spearman’s rank correlation coefficient. Additional Spearman rank correlations were used to assess concordance in seasonal, monthly, and hourly activity patterns, as well as in the temporal distribution of scent-marking behaviour.

Spatial structure in scent-marking behaviour was investigated separately for each species using Mantel tests and Moran’s I index. Mantel tests were used to evaluate the relationship between geographic distance among camera-trap stations and similarity in marking activity, based on distance matrices derived from station coordinates and marking frequencies. Moran’s index (I) was calculated to assess the presence of spatial autocorrelation in scent-marking activity across the study area.

For all analyses, statistical significance was set at α = 0.05.

## 3. Results

### 3.1. Study Design

The study was specifically designed to investigate behavioural dynamics of the European badger and the stone marten (Figure 3) during crepuscular–nocturnal period, when activity levels and scent-marking behaviour are most frequent for both species [26,27]. The decision to set camera traps from sunset to sunrise (7:00 p.m.–07:00 a.m.) was also influenced by the specific environmental context of the study area. Unlike a closed forest system, the “Calanchi di Atri” Reserve is characterized by open agro-pastoral habitats and ecotones subject to significant daytime human disturbance (tourists, hikers, farmers, and livestock herders), which can alter wildlife behaviour and increase non-target detections. Therefore, the camera trap scheduling was chosen to maximize reliable detections and minimize human interference and technical limitations.

Camera traps were positioned at an average height of 80–100 cm above ground level, a distance selected on the basis of the terrestrial habits of the European badger and the predominantly terrestrial habits of the stone marten. Although the latter is capable of climbing trees and occasionally using them, particularly for resting or accessing resources, its tendency to lead a predominantly terrestrial life compared to other mustelids (such as the pine marten) [36] suggests that this behaviour is likely limited within the study area, which is characterized primarily by low-growing vegetation and shrubs.

As information on the population density of the two species in the study area was not available, we decided to treat all records made at two-minute intervals as independent events in order to minimize data loss resulting from the inadvertent exclusion of legitimate subsequent visits by other individuals of the same species (e.g., a female following a male). When multiple markings were observed in one record, they were considered as a single event.

### 3.2. Spatial Distribution of European Badger and Stone Marten

During the camera-trapping monitoring period, a total of 765 detection events were recorded for the two carnivore species under study. *M. meles* was the most frequently detected species, with 595 events, whereas *M. foina* was recorded in 170 events. Consistent with the higher overall number of events, *M. meles* was generally recorded more frequently than *M. foina* at several camera-trap sites (Figure 4a). The highest numbers of badger detections were observed at camera traps D, E, and I, with site D showing the greatest number of records. In contrast, *M. foina* reached its highest detection frequency at camera trap I, showed moderate activity at site E, and was only occasionally detected or absent at the remaining stations. To account for the unequal detection frequencies between the two species, non-parametric rank-based analyses were performed using camera-trap stations as paired sampling units. The Wilcoxon signed-rank test revealed no significant differences in the proportional use of camera-trap stations between *M. meles* and *M. foina* (V = 37, *p* = 0.375), indicating similar relative spatial use across the monitored area.

The distribution of marking behaviour detections showed a similar pattern, but with lower absolute values (Figure 4b). Specifically, *M. meles* was observed engaging in marking behaviour in 203 events, whereas *M. foina* was recorded in 55 events. Camera traps D and E were the most relevant for *M. meles*, with a relatively high number of recorded events also at site I. In contrast, *M. foina* showed a very limited number of marking events, primarily concentrated at camera trap E and, to a lesser extent, at site I, with events absent or nearly absent at the other stations. Similarly to what was observed for detection frequencies, no significant differences emerged in the proportional distribution of scent-marking activity between the two species (V = 26, *p* = 0.722), suggesting no clear tendency to use different marking areas. Spearman rank correlations revealed positive associations in the spatial distribution of detections (rho = 0.644, *p* = 0.044) and marking events (rho = 0.650, *p* = 0.042) across camera-trap stations.

Spatial analyses based on camera trap coordinates did not reveal significant spatial autocorrelation for either species at the spatial scale and sampling resolution considered (Figure 5). For *M. meles,* the Mantel test showed a weak positive correlation (r = 0.219) which was not statistically significant (*p* = 0.165), indicating no clear relationship between geographic distance separating camera-trap stations and similarity in marking activity patterns. Likewise, *M. foina* exhibited a weak negative, highly non-significant correlation (r = −0.389; *p* = 0.948). These results were further supported by Moran’s I index, which did not detect significant spatial autocorrelation for either species (*M. meles*: observed = −0.093, expected = −0.111, *p* = 0.865; *M. foina*: observed = −0.217, expected = −0.167, *p* = 0.543).

### 3.3. Temporal Activity of European Badger and Stone Marten

To account for potential seasonal variation in activity rhythms, data analyses were stratified on a seasonal basis using the meteorological division of the year. Specifically, spring was defined as 1 March–31 May, summer as 1 June–31 August, autumn as 1 September–30 November, and winter as 1 December–28 February. This classification simplified calculations and more accurately reflected actual climatic periods.

The monthly pattern of observations revealed pronounced seasonality for both species. For *M. meles*, detection numbers were highest during winter and spring, with a pronounced peak in February and March, followed by still elevated values in April (Figure 6a). During summer, detections gradually decreased, reaching a minimum in August–September. In autumn, detections remained generally low. *M. foina* exhibited a partially overlapping seasonal pattern, although the overall number of detections was lower. Detections were most frequent in spring, peaking in April and remaining relatively high in May, whereas they decreased during summer and autumn, with the lowest values recorded between September and October.

For both species, the frequency of scent-marking events was higher during winter and spring. (Figure 6b). In *M. meles*, the number of marking events peaked between February and April, followed by a marked reduction during summer and generally low levels in autumn. In *M. foina*, marking events were considerably less frequent than in the European badger. The highest values were recorded in January and April, while during autumn, such behaviour was rare or absent.

Seasonal detection patterns, measured by kernel density estimation (Figure 7a), showed a positive correlation between the two species (rho = 0.870, *p* = 0.0002), indicating broadly similar seasonal trends despite differences in detection intensity. Similarly, monthly scent-marking frequency was positively correlated (rho = 0.679, *p* = 0.015), suggesting coincident seasonal peaks in marking behaviour (Figure 7b).

During the crepuscular–nocturnal period, differences in detection frequency and temporal distribution were observed between the two species (Figure 8a). *M. meles* exhibited higher detection rates between 8:00 p.m. and 11:59 p.m. A second peak in detection rate was also observed in the hours immediately after midnight, particularly between 1:00 and 1:59 a.m. In contrast, the detection rate was greatly reduced during the early morning hours, with the lowest values recorded between 5:00 and 6:59 a.m. *M. foina* showed a similar pattern, but with a much lower total number of detections. Records were mainly concentrated during the night-time period, with increased detections between 1:00 and 2:59 a.m. and between 9:00 and 11:59 p.m., while the lowest number of detections was recorded between 7:00 and 7:59 p.m.

As with the detection rate, the marking events of *M. meles* were mainly recorded during the late-night and evening hours, with higher frequencies coinciding with those observed in the detection rate and a minimum during the early morning hours (Figure 8b). For *M. foina*, the number of marking events was overall lower across all time intervals. Peaks occurred between 2:00 and 2:59 a.m. and between 10:00 and 10:59 p.m., while marking events were sporadic or absent during the central night-time and morning hours. In particular, very low values were observed between 6:00 and 6:59 a.m. and between 7:00 and 7:59 p.m.

Hourly patterns of camera-trap detections showed a non-significant positive association (rho = 0.496, *p* = 0.085), whereas hourly marking behaviour remained significantly correlated (rho = 0.568, *p* = 0.043), suggesting partially shared temporal windows for communication (Figure 9).

## 4. Discussion

By integrating results of temporal and spatial analyses, it was possible to obtain a more comprehensive understanding of how *M. meles* and *M. foina* coexist within the WWF “Calanchi di Atri” Regional Nature Reserve. Badgers and stone martens showed substantial spatial overlap, with only slight temporal and behavioural adjustments, suggesting that coexistence may be facilitated by fine-scale differences in temporal use and behaviour rather than by complete segregation [4,5].

Spatial analyses revealed variation in detection rates among camera-trap stations for both species, indicating that the use of the study area was not spatially uniform. Although *M. meles* was detected more frequently than *M. foina*, no clear evidence of spatial segregation emerged. Sites with the highest detection rates for one species also tended to have higher detection rates for the other, suggesting that both species may exploit similar habitat features or movement corridors. However, the limited number of spatial sampling units (10 camera traps) reduces the power to detect moderate or weak spatial patterns.

The two species showed partially overlapping nocturnal activity patterns [26,27,37,38,39]. The detection frequency of *M. meles* is higher during the central hours of the night, whereas *M. foina* showed a broader temporal distribution extending into crepuscular periods [40]. These differences may be correlated to differences in ecological strategies of the two species: badgers are strongly associated with established setts and well-defined movement routes within their territories [41,42], whereas stone martens exhibit greater flexibility in resource use and an opportunistic feeding strategy, adapting their diet to local and seasonal resource availability [43,44]. Subtle temporal differences may contribute to reducing direct encounters, despite the use of shared areas.

Seasonality appeared to be an important factor influencing activity. *M. meles* showed the highest detection frequencies during winter and spring, followed by a progressive decline during summer and autumn. *M. foina* exhibited a similar seasonal trend, although with lower overall activity levels. The strong seasonal correlation observed between species suggests a common response to environmental variations, likely driven by changes in resource availability and reproductive cycles.

Scent-marking behaviour provided additional insights into species ecology. *M. meles* showed a higher number of recorded marking events, particularly between late winter and spring, confirming the importance of chemical communication in territorial maintenance and social interactions. In contrast, scent-marking by *M. foina* was less frequent and more irregular, suggesting a less structured use of chemical signals. Nevertheless, both species exhibited similar seasonal trends in marking activity, with peaks corresponding to periods of reproductive and territorial activity.

Overall, the results suggest that *M. meles* and *M. foina* may slightly adjust their temporal patterns and communication behaviour, potentially facilitating the shared use of a restricted area. However, environmental and anthropogenic variables were not explicitly quantified or incorporated into the analyses, and their potential contribution to the observed patterns therefore remains to be assessed. Integrating behavioural information with factors such as habitat characteristics, resource availability, trophic interactions, and human disturbance may further refine this interpretation and provide additional insights into the ecological mechanisms underlying coexistence and communication behaviour in these mesocarnivores.

## 5. Conclusions

This study highlights how subtle adjustments in the timing of activity, seasonal variation, and distinct scent-marking behaviours may contribute to limiting potential interactions between *M. meles* and *M. foina* coexisting within a relatively restricted area. A more complete understanding of the mechanisms underlying their coexistence would require the integration of environmental variables, trophic data, and habitat use. In a heterogeneous and fragmented landscape such as the Calanchi di Atri, these mechanisms may contribute to reducing competition, allowing the two species to exploit available resources in a complementary manner.

## Figures and Tables

**Figure 1:**
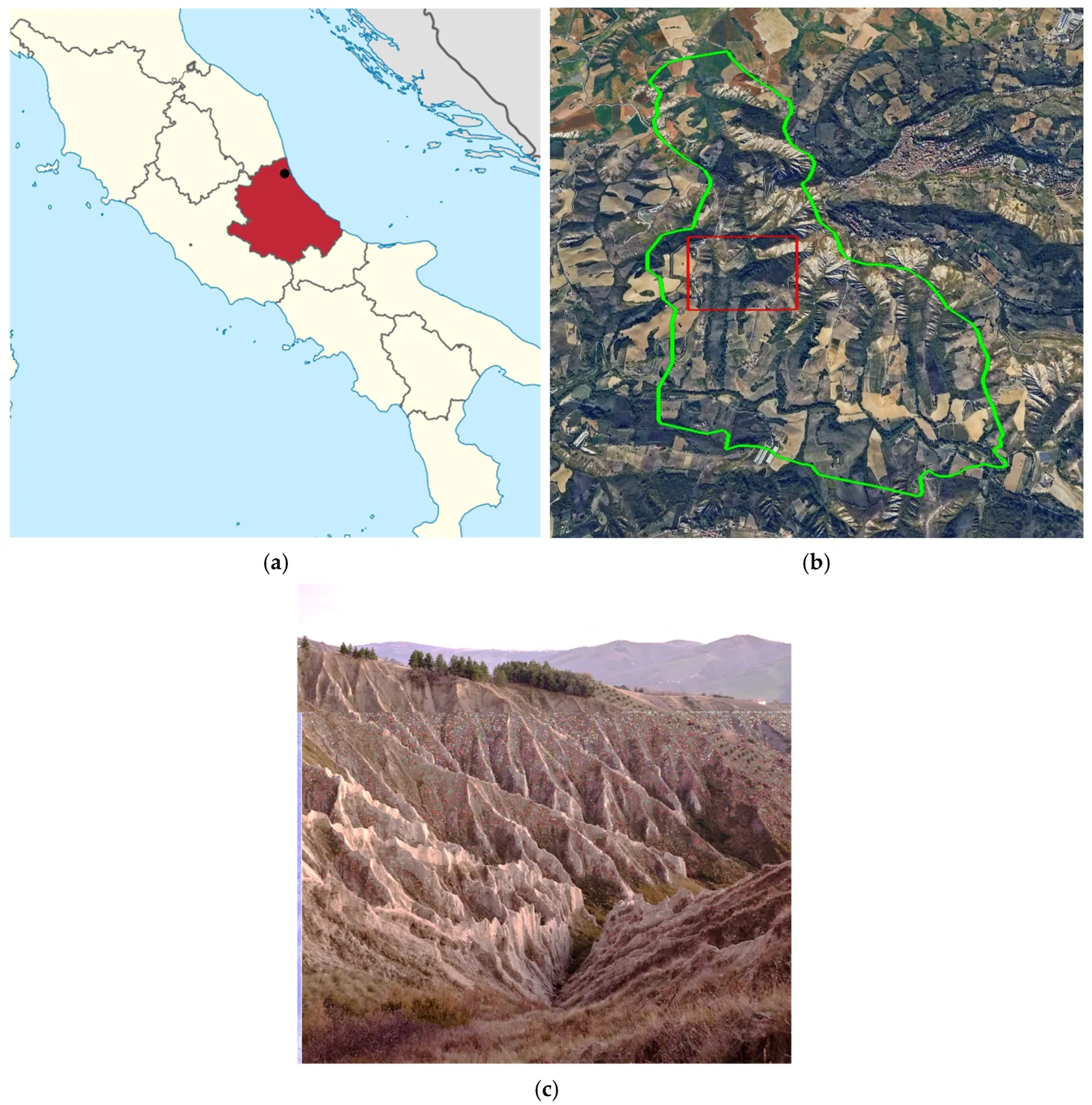
In the map of Italy (**a**), the Abruzzo region is shown in red, and the location of the Calanchi di Atri Regional Reserve is indicated by a black dot. Detailed view of the study area (**b**): boundaries of the Reserve are marked by a green line, and those of the study area by a red line. A view of the Atri badlands (**c**) (Photo by Matteo Ferretti).

**Figure 2:**
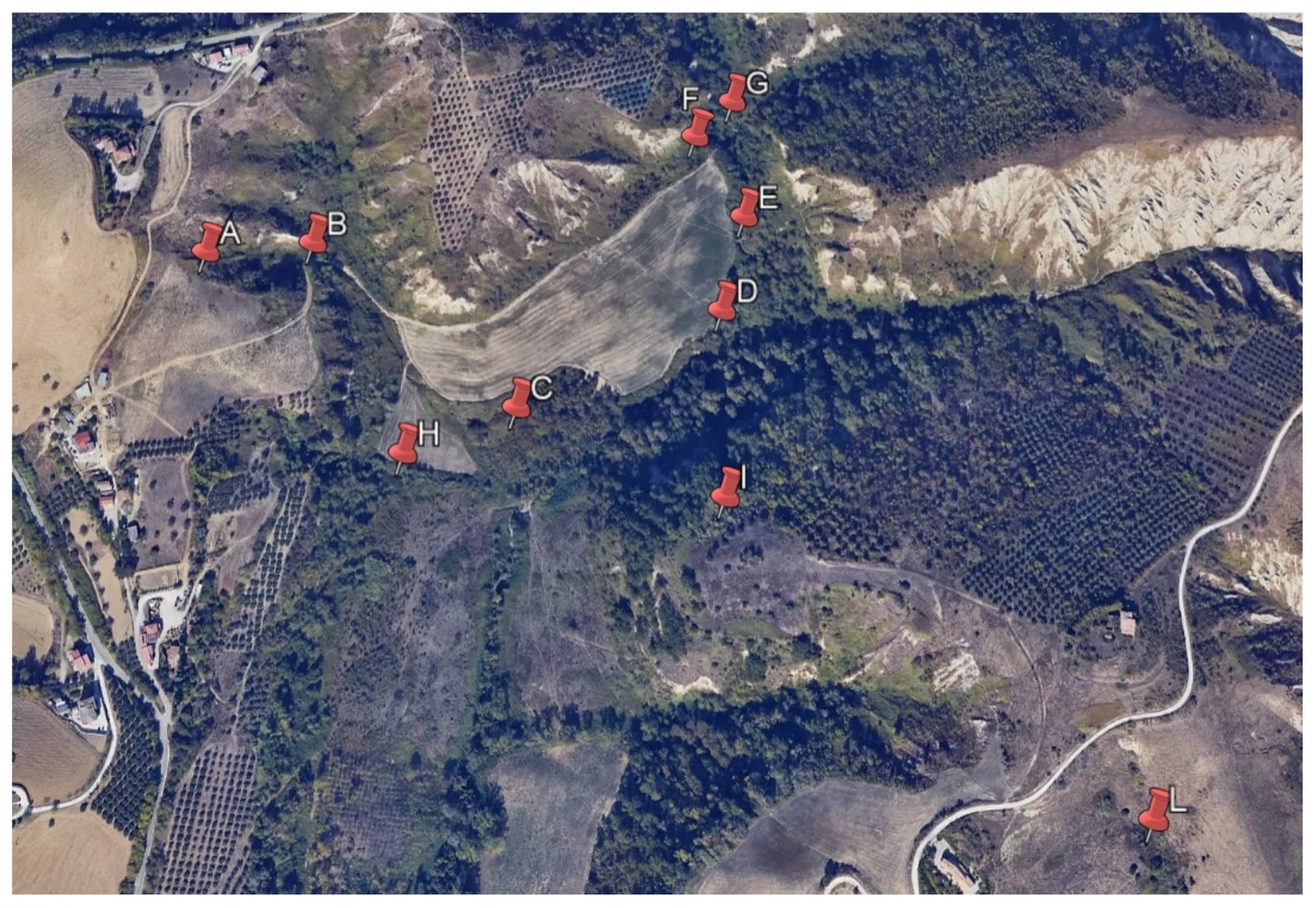
Map showing the locations of camera traps, indicated by red markers and letters.

**Figure 3:**
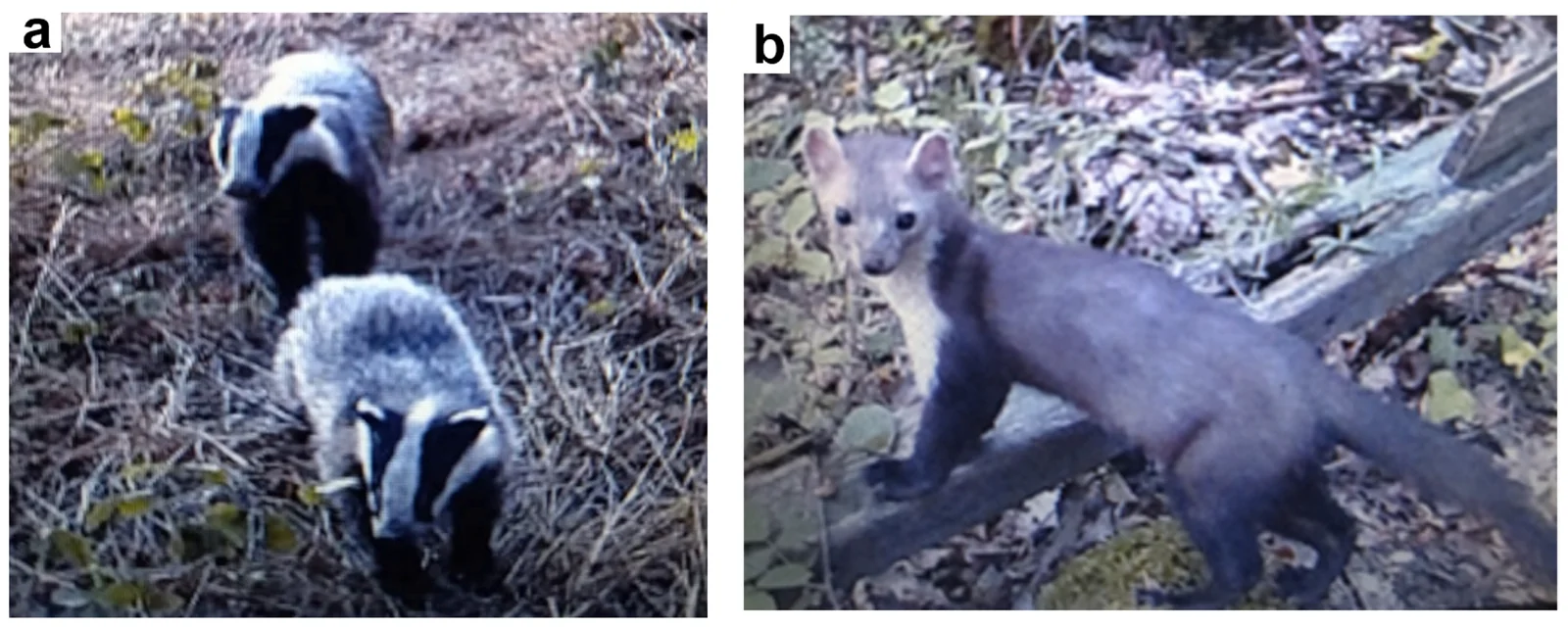
Two European badgers (**a**) and one stone marten (**b**) recorded by a camera trap used in this study.

**Figure 4:**
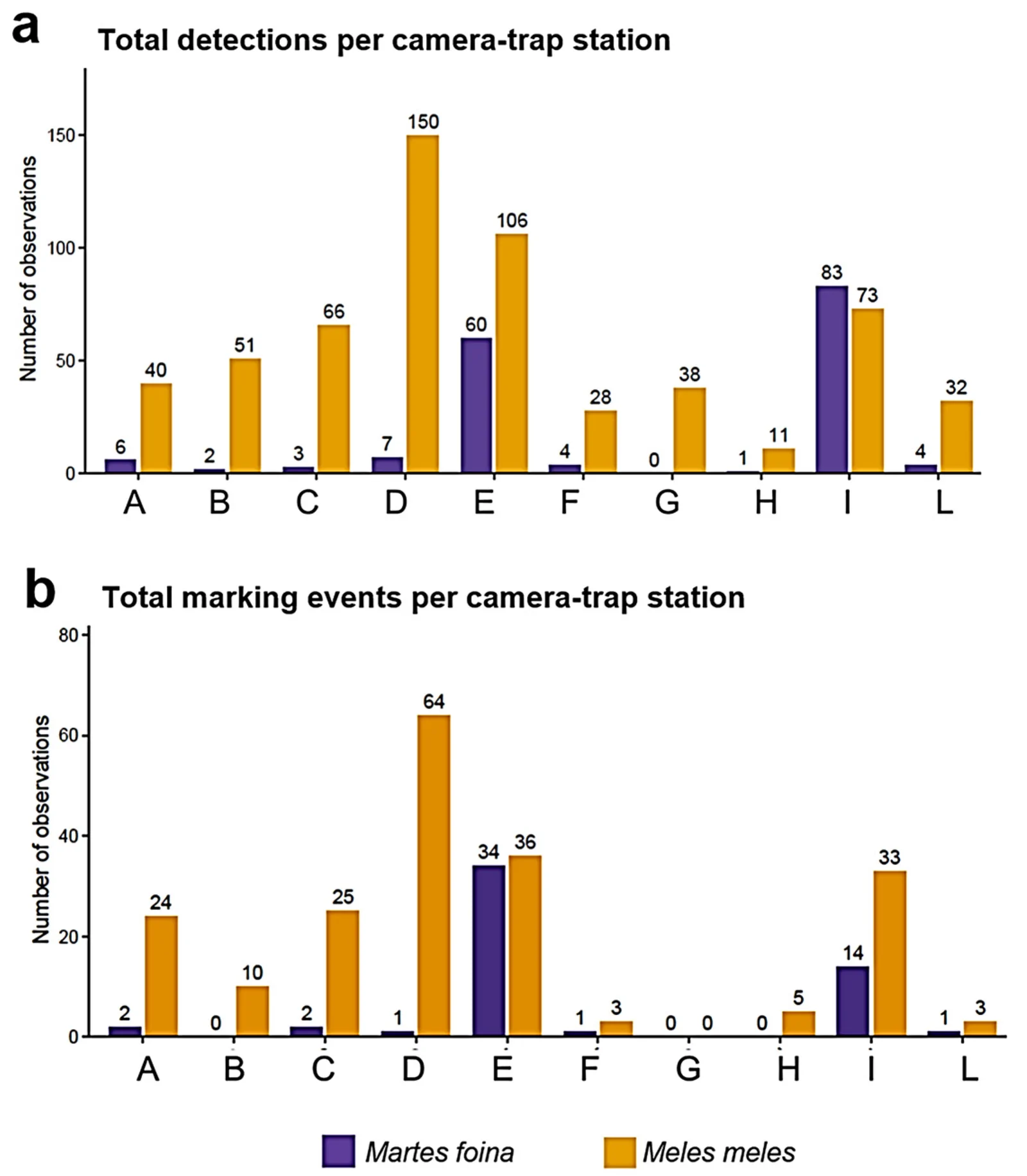
Number of detections per camera-trap station of European badgers and stone martens (**a**), and number of detections of the two species during marking activity (**b**). Camera trap stations are indicated by letters as in Figure 2.

**Figure 5:**
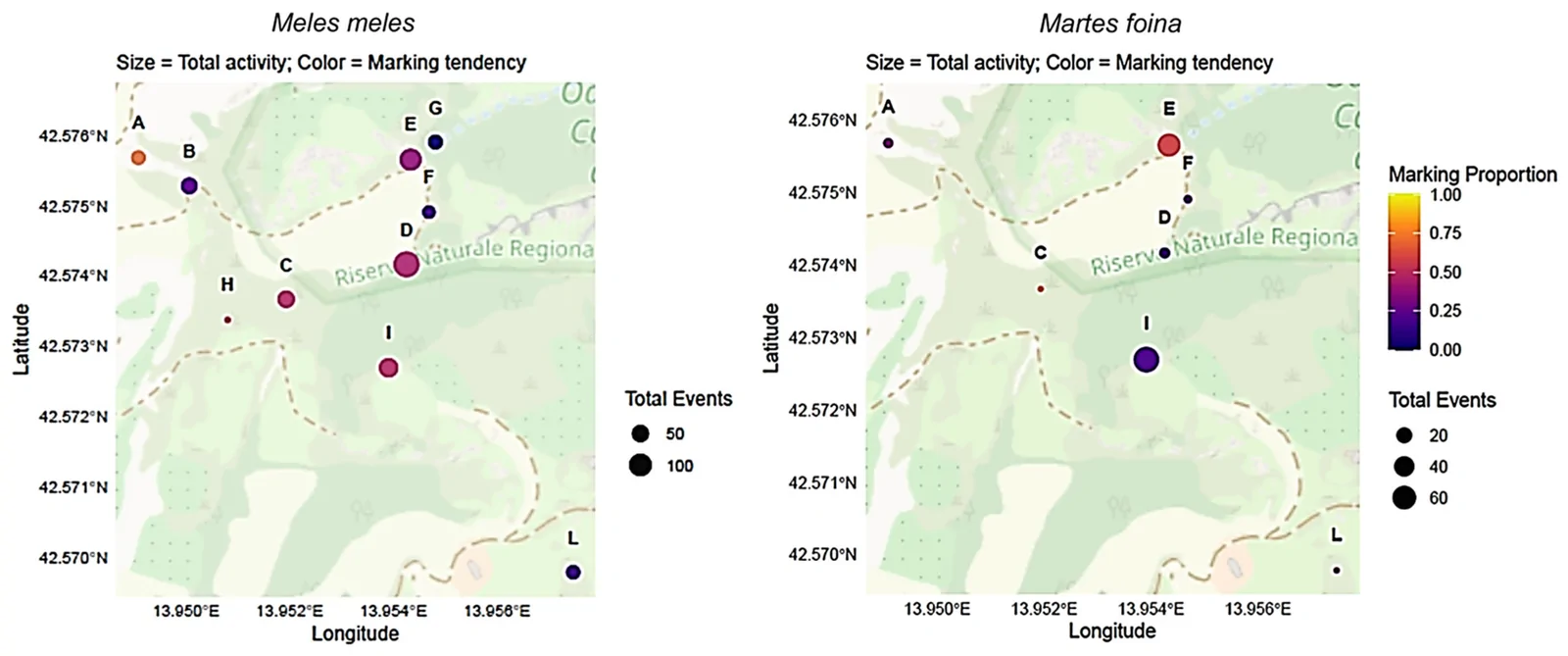
Mantel test relating geographic distance separating camera-trap sites to differences in scent-marking activity of *M. meles* and *M.s foina*. Camera trap stations are indicated by letters as in Figure 2.

**Figure 6:**
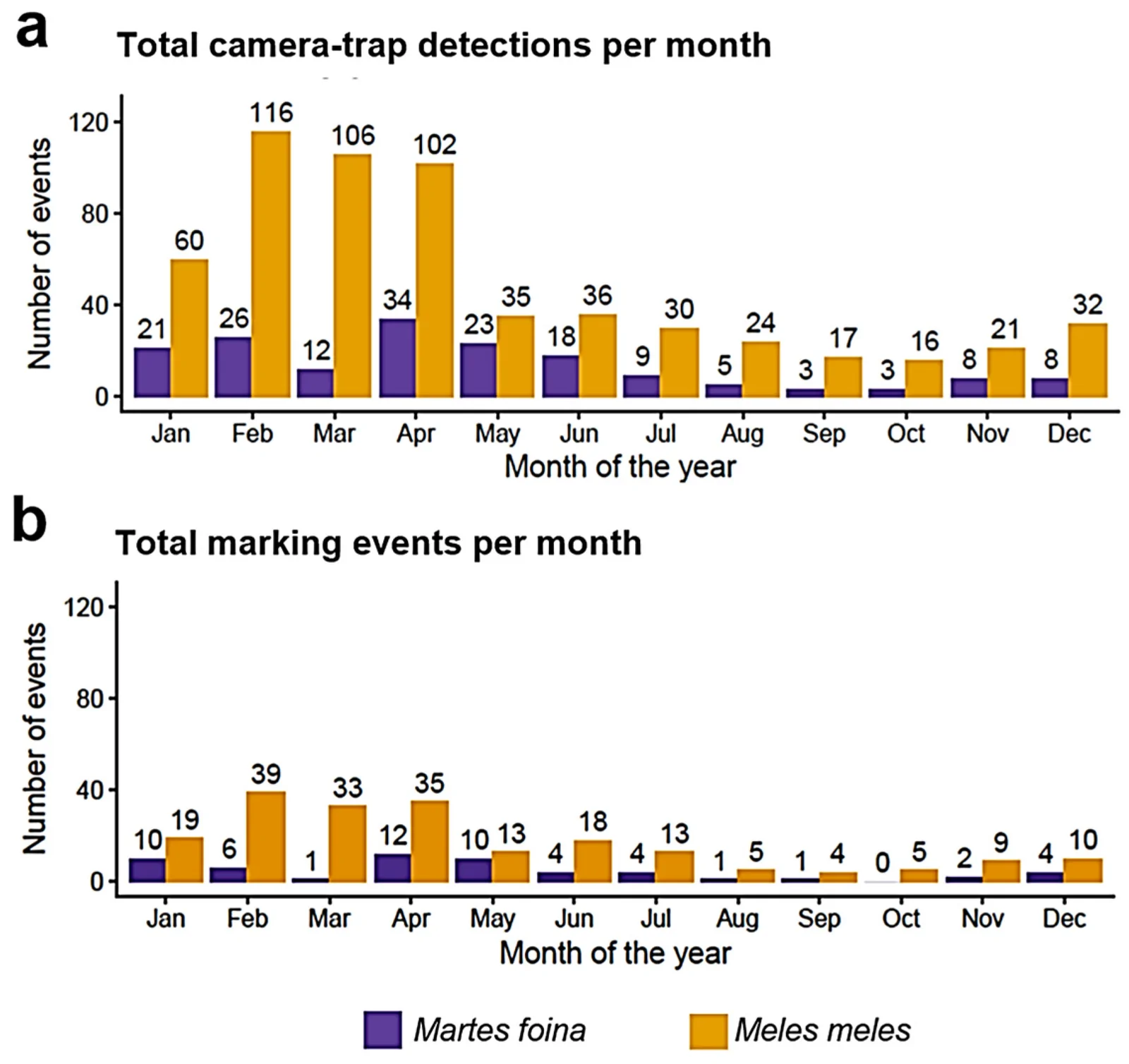
Monthly number of camera-trap detections of European badgers and stone martens (**a**) and detections of the two species during scent-marking behaviour (**b**).

**Figure 7:**
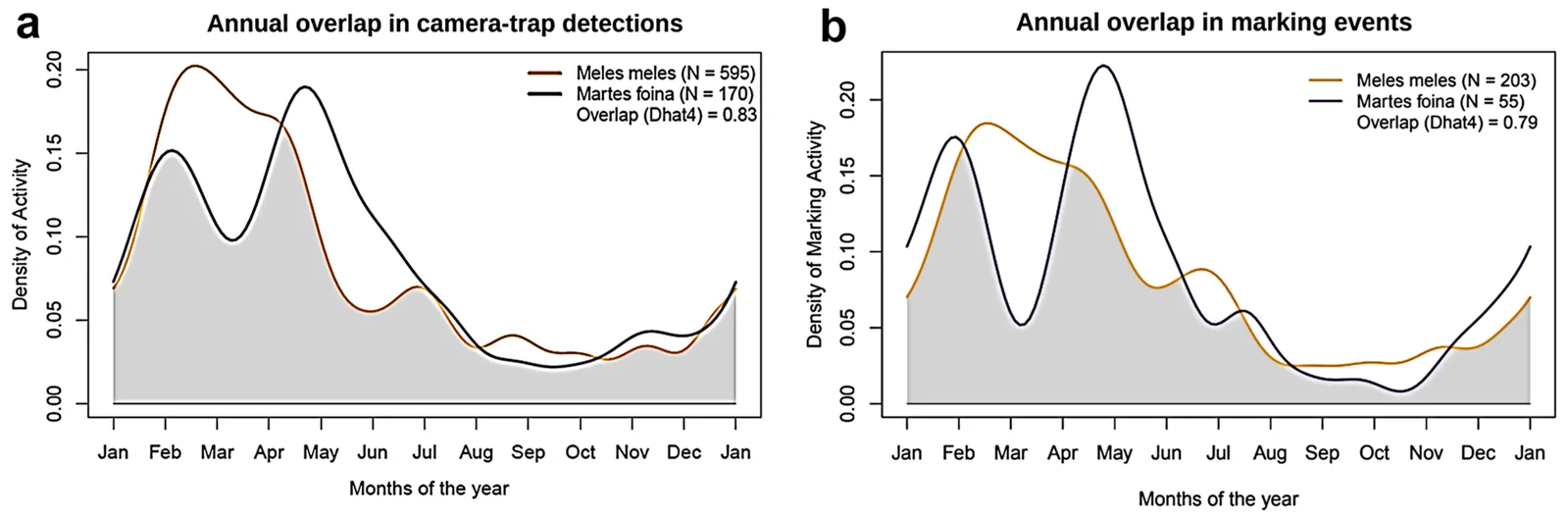
Annual overlap in camera-trap detections (**a**) and marking events (**b**) of European badgers and stone martens. Curves represent kernel density values based on daily records and are standardized so that the area under each curve equals 1, thereby describing the temporal distribution of records throughout the year rather than absolute record levels. The overlap coefficient (Dhat4) quantifies the degree of temporal overlap between species, ranging from 0 (no overlap) to 1 (complete overlap). The grey area indicates the overlap between the activity patterns of *M. meles* and *M. foina*.

**Figure 8:**
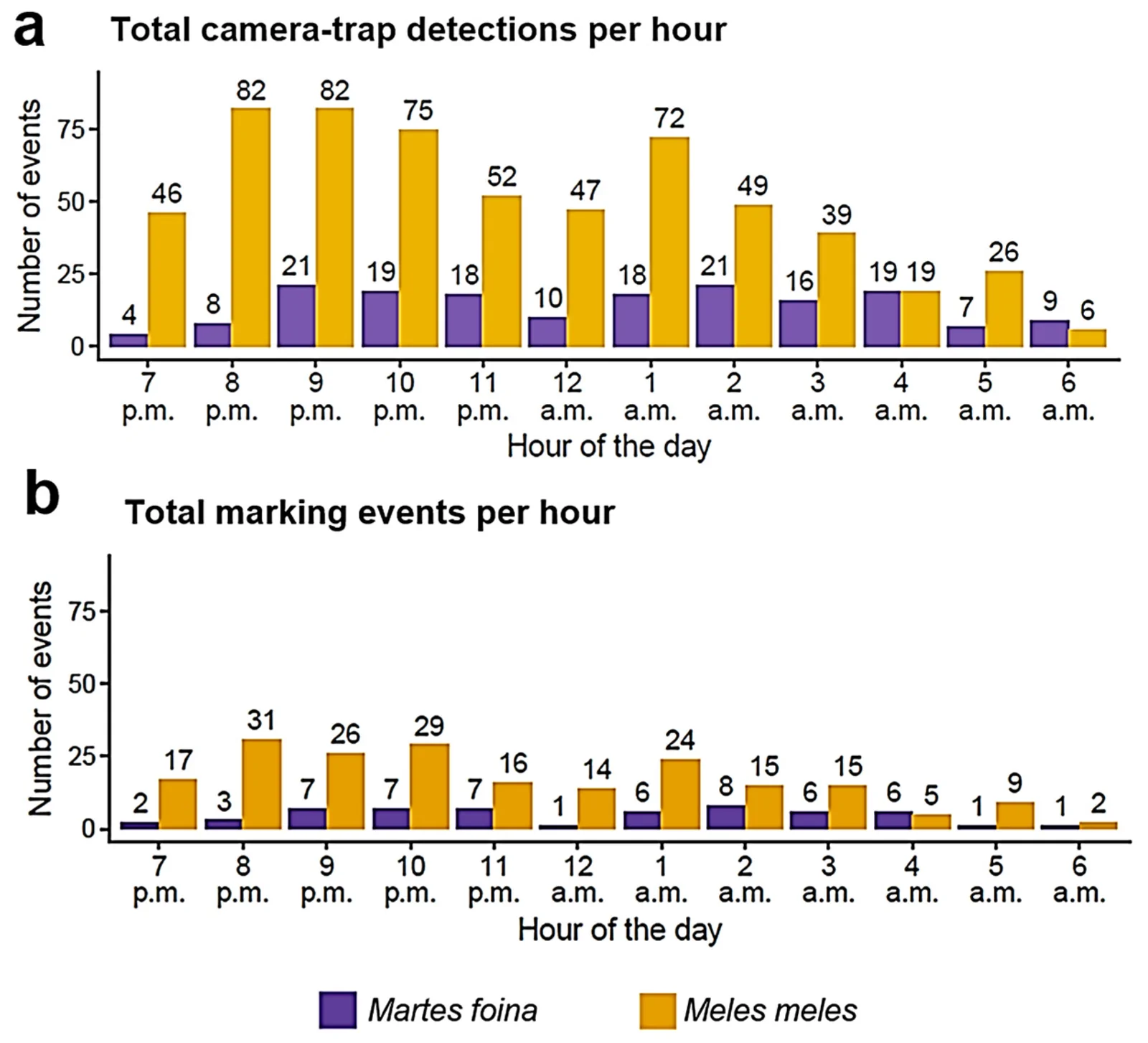
Hourly distribution of camera-trap detections of European badgers and stone martens (**a**) and detections of the two species during scent-marking activity (**b**).

**Figure 9:**
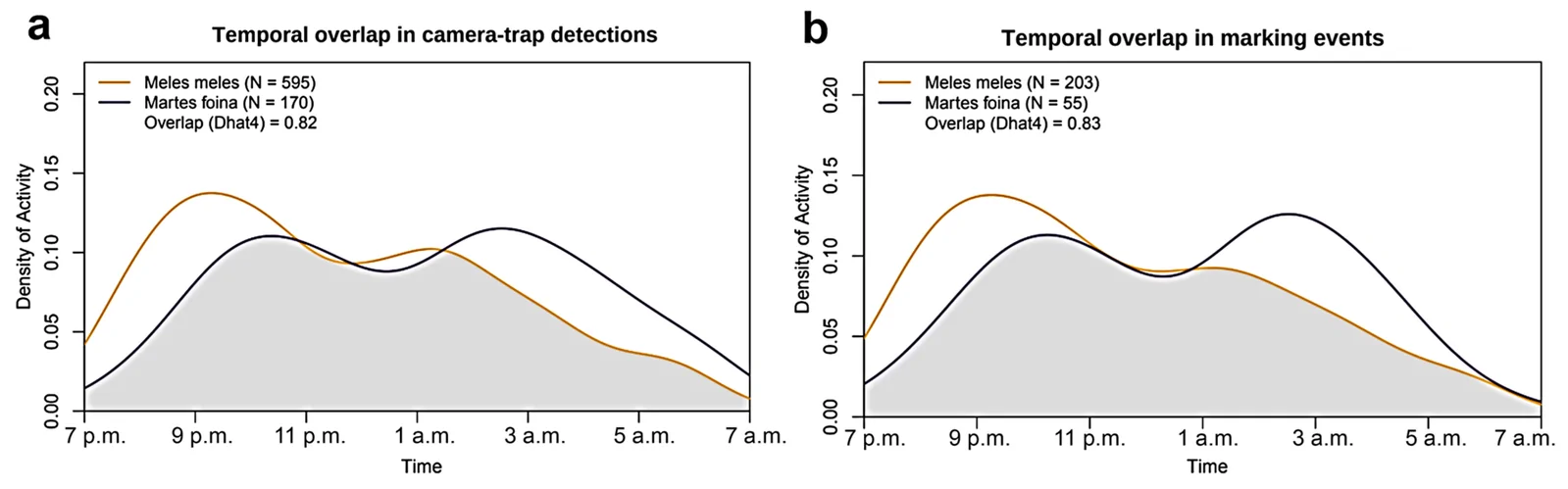
Temporal overlap in camera-trap detections (**a**) and marking events (**b**) of European badgers and stone martens at the indicated times. Curves represent kernel density estimates derived from hourly records and standardized to unit area, thereby illustrating the relative distribution over time rather than absolute frequencies. The overlap coefficient (Dhat4) quantifies the degree of temporal overlap between species, ranging from 0 (no overlap) to 1 (complete overlap). The grey area indicates the overlap between the temporal patterns of *M. meles* and *M. foina*.

## Data Availability

The original contributions presented in this study are included in the article material. Further inquiries can be directed to the corresponding authors.

## References

[1] Fisher J.T., Wilkinson L. (2005). The response of mammals to forest fire and timber harvest in the North American boreal forest. Mammal Rev..

[2] Ridout M.S., Linkie M. (2009). Estimating overlap of daily activity patterns from camera trap data. J. Agric. Biol. Environ. Stat..

[3] Mori E., Bagnato S., Serroni P., Sangiuliano A., Rotondaro F., Marchianò V., Cascini V., Poerio L., Ferretti F. (2019). Spatiotemporal mechanisms of coexistence in an European mammal community in a protected area of southern Italy. J. Zoöl..

[4] Ferreiro-Arias I., Isla J., Jordano P., Benítez-López A. (2021). Fine-scale coexistence between Mediterranean mesocarnivores is mediated by spatial, temporal, and trophic resource partitioning. Ecol. Evol..

[5] Monterroso P., Alves P.C., Ferreras P. (2014). Plasticity in circadian activity patterns of mesocarnivores in Southwestern Europe: Implications for species coexistence. Behav. Ecol. Sociobiol..

[6] Rossa M., Lovari S., Ferretti F. (2021). Spatiotemporal patterns of wolf, mesocarnivores and prey in a Mediterranean area. Behav. Ecol. Sociobiol..

[7] Linck P., Palomares F., Negrões N., Rossa M., Fonseca C., Couto A., Carvalho J. (2023). Increasing homogeneity of Mediterranean landscapes limits the co-occurrence of mesocarnivores in space and time. Landsc. Ecol..

[8] Genovesi P., Boitani L. (1995). Preliminary data on the social ecology of the stone marten (*Martes foina* Erxleben 1777) in Tuscany (Central Italy). Hystrix Ital. J. Mammal..

[9] Kowalczyk R., Jȩdrzejewska B., Zalewski A. (2003). Annual and circadian activity patterns of badgers (*Meles meles*) in Białowieża Primeval Forest (eastern Poland) compared with other Palaearctic populations. J. Biogeogr..

[10] Kowalczyk R., Zalewski A., Bogumiła J. (2006). Daily movement and territory use by badgers *Meles meles* in Białowieża Primeval Forest, Poland. Wildl. Biol..

[11] López-Martin J.M., Santos-Reis M., Birks J.D.S., O’Doherty E.C., Proulx G. (2006). Comparison of feeding behaviour between stone marten and common genet: Living in coexistence. Martes in Carnivore Communities.

[12] Santos M.J., Santos-Reis M. (2009). Stone marten (*Martes foina*) habitat in a Mediterranean ecosystem: Effects of scale, sex, and interspecific interactions. Eur. J. Wildl. Res..

[13] Wereszczuk A., Leblois R., Zalewski A. (2017). Genetic diversity and structure related to expansion history and habitat isolation: Stone marten populating rural–urban habitats. BMC Ecol..

[14] Roper T.J. (1992). Badger *Meles meles* setts–architecture, internal environment and function. Mammal Rev..

[15] Buesching C.D., Macdonald D.W., Marchlewska-Koj A., Lepri J.J., Müller-Schwarze D. (2001). Scent-marking behaviour of the European badger (*Meles meles*): Resource defence or individual advertisement?. Chemical Signals in Vertebrates 9.

[16] Genovesi P., Sinibaldi I., Boitani L. (1997). Spacing patterns and territoriality of the stone marten. Can. J. Zoöl..

[17] Balestrieri A., Mosini A., Fonda F., Piana M., Tirozzi P., Ruiz-González A., Capelli E., Vergara M., Chueca L.J., Chiatante G. (2021). Spatial ecology of the stone marten in an Alpine area: Combining camera-trapping and genetic surveys. Mammal Res..

[18] Macdonald D.W., Buesching C.D., Stopka P., Henderson J., Ellwood S.A., Baker S.E. (2004). Encounters between two sympatric carnivores: Red foxes (*Vulpes vulpes*) and European badgers (*Meles meles*). J. Zoöl..

[19] Yamaguchi N., Dugdale H.L., Macdonald D.W. (2006). Female Receptiveity, Embryonic Diapause, and Superfetation in the European Badger (*Meles Meles*: Implications for the Reproductive Tactics of Males and Females. Q. Rev. Biol..

[20] Corner L.A.L., Stuart L.J., Kelly D.J., Marples N.M. (2015). Reproductive Biology Including Evidence for Superfetation in the European Badger *Meles meles* (Carnivora: Mustelidae). PLoS ONE.

[21] Sugianto N. (2021). Reproductive Biology of the European Badger (*Meles meles*): Endocrinological Insights into Lifetime Reproductive Events, Strategies and Cub Development in Response to Ecological Factors. Ph.D. Thesis.

[22] Canivenc R., Mauget C., Bonnin M., Aitken R.J. (1981). Delayed implantation in the Beech marten (*Martes foina*). J. Zoöl..

[23] Herr J., Schley L., Roper T.J. (2009). Socio-spatial organization of urban stone martens. J. Zoöl..

[24] Steklenev E.P. (2014). Morphological and Physiological Characteristics of Reproduction of the Stone Marten, *Martes foina* (Mammalia, Carnivora), in the Steppe Zone of the South of Ukraine. Zoodiversity.

[25] Balestrieri A., Ruiz-González A., Capelli E., Vergara M., Prigioni C., Saino N. (2016). Pine marten vs. stone marten in agricultural lowlands: A landscape-scale, genetic survey. Mammal Res..

[26] Torretta E., Mosini A., Piana M., Tirozzi P., Serafini M., Puopolo F., Saino N., Balestrieri A. (2017). Time partitioning in mesocarnivore communities from different habitats of NW Italy: Insights into martens’ competitive abilities. Behaviour.

[27] Bisi F., Grespan P., Tranquillo C., Martinoli A., Wauters L.A., Preatoni D.G. (2025). Year-Round Activity Patterns of Badgers (*Meles meles*) and Mesocarnivore Communities in Urban and Sub-Urban Areas. Urban Sci..

[28] Buesching C.D., Jordan N. (2019). The social function of latrines: A hypothesis-driven research approach. Chemical Signals in Vertebrates 14.

[29] Burton A.C., Neilson E., Moreira D., Ladle A., Steenweg R., Fisher J.T., Bayne E.M., Boutin S. (2015). REVIEW: Wildlife camera trapping: A review and recommendations for linking surveys to ecological processes. J. Appl. Ecol..

[30] Rovero F., Zimmermann F. (2016). Camera features related to specific ecological applications. Camera Trapping for Wildlife Research.

[31] Steenweg R., Hebblewhite M., Kays R., Ahumada J., Fisher J.T., Burton C., Townsend S.E., Carbone C., Rowcliffe J.M., Whittington J. (2017). Scaling-up camera traps: Monitoring the planet’s biodiversity with networks of remote sensors. Front. Ecol. Environ..

[32] Caravaggi A., Banks P.B., Burton A.C., Finlay C.M.V., Haswell P.M., Hayward M.W., Rowcliffe M.J., Wood M.D. (2017). A review of camera trapping for conservation behaviour research. Remote Sens. Ecol. Conserv..

[33] Torretta E., Serafini M., Puopolo F., Schenone L. (2016). Spatial and temporal adjustments allowing the coexistence among carnivores in Liguria (N-W Italy). Acta Ethologica.

[34] Reyserhove L., Norton B., Desmet P. (2023). Best Practices for Managing and Publishing Camera Trap Data.

[35] Niedballa J., Sollmann R., Courtiol A., Wilting A. (2016). camtrapR: An R package for efficient camera trap data management. Methods Ecol. Evol..

[36] Sacchi O., Meriggi A. (1995). Habitat requirements of the stone marten (*Martes foina*) on the Tyrrhenian slopes of the northern Apennines. Hystrix Ital. J. Mammal..

[37] Palencia P., Fernández-López J., Vicente J., Acevedo P. (2021). Innovations in movement and behavioural ecology from camera traps: Day range as model parameter. Methods Ecol. Evol..

[38] Vilella M., Ferrandiz-Rovira M., Sayol F. (2020). Coexistence of predators in time: Effects of season and prey availability on species activity within a Mediterranean carnivore guild. Ecol. Evol..

[39] Mori E., Menchetti M. (2019). Living with roommates in a shared den: Spatial and temporal segregation among semifossorial mammals. Behav. Process..

[40] Tsunoda H., Newman C., Peeva S., Raichev E., Buesching C.D., Kaneko Y. (2020). Spatio-temporal partitioning facilitates mesocarnivore sympatry in the Stara Planina Mountains, Bulgaria. Zoology.

[41] Revilla E., Palomares F. (2002). Spatial organization, group living and ecological correlates in low-density populations of Eurasian badgers, *Meles meles*. J. Anim. Ecol..

[42] Reid N., Etherington T.R., Wilson G.J., Montgomery W.I., McDonald R.A. (2012). Monitoring and population estimation of the European badger *Meles meles* in Northern Ireland. Wildl. Biol..

[43] Bakaloudis D.E., Vlachos C.G., Papakosta M.A., Bontzorlos V.A., Chatzinikos E.N. (2012). Diet Composition and Feeding Strategies of the Stone Marten (*Martes foina*) in a Typical Mediterranean Ecosystem. Sci. World J..

[44] Balestrieri A., Remonti L., Capra R.B., Canova L., Prigioni C. (2013). Food habits of the stone marten (*Martes foina*) (Mammalia: Carnivora) in plain areas of Northern Italy prior to pine marten (*M. martes*) spreading. Ital. J. Zoöl..

